# Strategies for implementing genomic selection in family-based aquaculture breeding schemes: double haploid sib test populations

**DOI:** 10.1186/1297-9686-44-30

**Published:** 2012-10-30

**Authors:** Kahsay G Nirea, Anna K Sonesson, John A Woolliams, Theo HE Meuwissen

**Affiliations:** 1 Department of Animal and Aquacultural Sciences, Norwegian University of Life Science, P.O. Box 5003, Ås, 1432, Norway; 2 Nofima AS, P.O. Box 210, Ås, 1431, Norway; 3 The Roslin Institute and Royal (Dick) School of Veterinary Studies, University of Edinburgh, Easter Bush Campus, Midlothian, EH25 9RG, Scotland, UK

## Abstract

**Background:**

Simulation studies have shown that accuracy and genetic gain are increased in genomic selection schemes compared to traditional aquaculture sib-based schemes. In genomic selection, accuracy of selection can be maximized by increasing the precision of the estimation of SNP effects and by maximizing the relationships between test sibs and candidate sibs. Another means of increasing the accuracy of the estimation of SNP effects is to create individuals in the test population with extreme genotypes. The latter approach was studied here with creation of double haploids and use of non-random mating designs.

**Methods:**

Six alternative breeding schemes were simulated in which the design of the test population was varied: test sibs inherited maternal (*Mat*), paternal (*Pat*) or a mixture of maternal and paternal (*MatPat*) double haploid genomes or test sibs were obtained by maximum coancestry mating (*MaxC*), minimum coancestry mating (*MinC*), or random (*RAND*) mating. Three thousand test sibs and 3000 candidate sibs were genotyped. The test sibs were recorded for a trait that could not be measured on the candidates and were used to estimate SNP effects. Selection was done by truncation on genome-wide estimated breeding values and 100 individuals were selected as parents each generation, equally divided between both sexes.

**Results:**

Results showed a 7 to 19% increase in selection accuracy and a 6 to 22% increase in genetic gain in the *MatPat* scheme compared to the *RAND* scheme. These increases were greater with lower heritabilities. Among all other scenarios, i.e. *Mat, Pat, MaxC*, and *MinC*, no substantial differences in selection accuracy and genetic gain were observed.

**Conclusions:**

In conclusion, a test population designed with a mixture of paternal and maternal double haploids, i.e. the *MatPat* scheme, increases substantially the accuracy of selection and genetic gain. This will be particularly interesting for traits that cannot be recorded on the selection candidates and require the use of sib tests, such as disease resistance and meat quality.

## Background

In traditional aquaculture breeding schemes, selection for traits that cannot be measured on the selection candidates (e.g. disease resistance and fillet quality) is based on a performance test of sibs of the candidates, i.e. information on test sibs is used to calculate breeding values for the selection of parents. This is due to the fact that measuring meat quality traits requires killing of the fish and fish that have been challenge-tested for disease resistance cannot be used as breeding stock. However, with a sib test, only 50% of the total genetic variance of the candidates is exploited, perhaps less. Recently, with the advent of high-throughput genotyping of genetic markers, genomic selection [1] has been taken up by animal breeders. With genomic selection, the total genetic value of the selection candidates is predicted based on the simultaneous estimation of single nucleotide polymorphism (SNP) effects using a set of individuals that have been genotyped and phenotyped [1]. Compared to traditional genetic evaluation methodologies and marker-assisted selection, genomic selection can result in an increase in the accuracy of selection for an individual without a phenotype provided that the test set is sufficiently large and relevant to the selected population [2]. Genomic selection is increasingly used in dairy cattle breeding [3-5] and in plant breeding [6,7] but is not yet used in selective breeding in aquaculture.

Simulation studies have examined possible strategies to implement genomic selection in family-based aquaculture breeding schemes, generally following two stages [8]: first, SNP marker effects are estimated in a test population consisting of sibs of the selection candidates and second, genome-wide breeding values of the genotyped selection candidates are estimated by summing up the estimated SNP marker effects. The benefits reported from these strategies are promising. In one study [8], in which sibs were performance-tested every generation, the accuracy of the estimated breeding values of selection candidates increased, which increased genetic gain because genetic gain is directly related to the accuracy of selection. In addition, genomic selection more than halved the rate of inbreeding compared to traditional BLUP selection using similar resources. A major contributor to these results is the accurate estimation of the within-family variance with genomic selection. Other studies have supported these findings [9].

Two factors are important for maximizing the accuracy of genomic breeding values: (1) increasing the precision of estimates of SNP marker effects in the test population; this can be achieved by increasing the number of animals in the test population and by increasing the number of SNP markers sufficiently to capture the genetic variance throughout the genome [10,11]; (2) maximising the relationship between individuals in the test and candidate populations [12].

Another means of increasing the accuracy of estimates of SNP effects is to have the test population consist of extreme genotypes. This approach has been exploited with the use of double haploids for QTL mapping in fish [13]. Double haploids are homozygous for all loci and thus achieve in a single generation more homozygosity than 10 generations of continuous full-sib mating [14]. Double haploids are produced by chromosome manipulation techniques such as gynogenesis and androgenesis, which produce female and male double haploids, respectively. With both these techniques, the duplicated chromosomes can be combined either before (mitotic) or after recombination (meiotic). Although the availability of double haploids is a major advantage in fish breeding, a number of drawbacks have also been reported, including technological challenges, costs of implementation, and the low viability of the progeny due to inbreeding depression [15]. An alternative approach to increasing homozygosity is to use non-random mating designs such as maximum coancestry mating.

Based on these considerations, it is hypothesized that designing a test population using double haploids or non-random mating can increase the accuracy of estimates of SNP effects in test sibs, which in turn will increase the accuracy of predicted breeding values when applied in genomic selection schemes. Given the reliance of many aquaculture schemes on sib testing, this hypothesis was tested by simulating a typical breeding scheme in fish.

## Methods

Simulation of populations was carried out in two steps: (1) to create base populations (G0) with a set of genomic data and (2) to simulate breeding schemes derived from these base populations. Details are presented in the following section.

### Simulation of the base population (generation G0)

We simulated a Fisher-Wright population with an effective population size of 1000 (500 males and 500 females) for 4000 generations to construct the base population G0. Four thousand generations has been shown to be sufficient to achieve mutation-drift equilibrium and stationary distributions of pair-wise linkage disequilibrium [8]. Within each of these generations, 500 males and 500 females were produced by random selection and mating of a sire and dam, with replacement after each mating.

A diploid genome with 10 chromosomes of 1 Morgan (M) each was simulated. SNP mutations and recombinations were introduced every generation at a rate of 10^-8^ per base pair per meiosis, assuming 10^8^ base pairs per M, which is close to the infinite sites mutation model [16]. SNP were passed from parent to offspring following Mendelian inheritance and recombination followed the Haldane mapping function [17].

After 4000 generations, the G0 generation was created with N_m_ = 3000 and N_f_ = 3000 offspring obtained from the random mating of *n*_*s*_ = 50 sires and n_*d*_ = 50 dams with replacement. To obtain a reliable result, the base population was replicated 100 times. For each base population, six different breeding schemes were run. The average of these replicates was used for comparison of the breeding schemes. Quantitative SNP effects were simulated to attribute breeding values to each individual for the trait evaluated in the sib test, as described in the following section.

### Simulation of generations for breeding schemes

Males and females from each G0 family were equally divided to create a test population and a candidate population, each with 3000 individuals. The phenotypes and genotypes of the test population were then evaluated using genomic evaluation techniques to estimate the SNP effects. Genomic estimated breeding values (EBV) for the candidates were computed based on their genotypes and these estimates. The best *n*_*s*_ = 50 male and *n*_*d*_ = 50 female candidates were selected to be sires and dams based on the EBV and randomly mated in pairs with *n*_*o*_ = 120 offspring/pair to produce generation G1 with 6000 offspring. Generation G0 also acted as the base population for pedigree numerator relationships.

In G1, the procedure for G0 was repeated up to the point of mating. The offspring were divided into test and candidate populations. Genomic evaluation was carried out using the phenotypes of the test population to estimate SNP effects; the best *n*_*s*_ = 50 male and *n*_*d*_ = 50 female candidates were then selected. In the genomic evaluation of G1, the accumulated data from both G0 and G1 test populations were used to estimate SNP effects.

The selected males and females in G1 were then used to produce a candidate population and a test population in G2. The mating of the G2 individuals defined the different breeding schemes of the study. For all schemes, the 3000 individuals from the G2 candidate population were created by pair-wise random mating among the *n*_*s*_ = 50 sires and *n*_*d*_ = 50 dams. However, individuals in the test population were created either as diploids following random mating, or following minimum or maximum coancestry mating, or as double haploids as described in the following section. Finally, in G2, SNP effects were re-estimated using all the accumulated test data from G0, G1, and G2. Then, the genomic EBV of the candidates were calculated and comparisons among the different breeding schemes were made.

### Deriving alternative test populations in G2

Two types of approaches were adopted to design the test population in G2: a diploid approach in which mating among G1 parents was managed, and a double haploid approach following the random mating among the G1 parents.

Three diploid approaches were simulated as follows:

Random mating (RAND)

Mating pairs were chosen from the *n*_*s*_ and *n*_*d*_ selected parents using random sampling without replacement to produce the G2 test population. Mating pairs were re-sampled from the same sets of parents to produce the G2 candidate population. This was done to allow fair comparisons with the assortative mating schemes described below.

Maximum coancestry (MaxC)

Using the selected males and females, mating pairs were chosen to maximize the average coancestry of the mates based on pedigree. First, a matrix of coancestries for all possible matings was constructed. Starting from an initial set of mating pairs, two pairs were chosen at random and their mates swapped. If this resulted in an increase in average coancestry, the swap was accepted, otherwise it was rejected. This was repeated until no further improvement was obtained.

Minimum coancestry (MinC)

Minimum coancestry mating was the same as maximum coancestry mating, except that mating pairs were designed to minimize average coancestry among the set of mates as in [18].

Three double haploid approaches were simulated. For all three approaches, the parents and mating pairs that created the G2 test and candidate populations were the same randomly selected group. In *MatPat,* 1500 progeny each were created by mitotic androgenesis from the G1 male parents, and by mitotic gynogenesis from the G1 female parents. Each parent produced 30 double haploid offspring. In *Pat*, all 3000 progeny came from the 50 male G1 parents by mitotic androgenesis, with 60 offspring per parent and no contribution from the female G1 parents. In *Mat*, G1 female parents contributed all the offspring by mitotic gynogenesis in a similar fashion as *Pat.*

### Simulation of markers, quantitative trait loci and true breeding values

In G0, a random sample of 1000 SNP from among those with a minor allele frequency (MAF) greater than 0.05 were assigned to be QTL. Additive allelic values were assigned to each QTL by independent sampling of effects from a Laplace distribution. True breeding values were computed as the sum of allelic effects at the 1000 QTL as follows:

(1) $$TBV_{i}=\sum_{J=1}^{1000}z_{ij1}g_{j1}+z_{ij2}g_{j2}$$

where *z*_*ijk*_ is the number of copies of allele *k* (*k* = 0, 1 or 2) at the *j*^*th*^ QTL locus of individual *i* and *g*_*jk*_ is the sampled effect. Allelic effects were then scaled to set the total genetic variance (*σ*_*A*_^2^) observed in G0 equal to 10.

Phenotypes with a heritability of 0.05, 0.1 or 0.4 were created by sampling environmental deviations from appropriate normal distributions and added these to the true breeding value. The *m* = 5000 SNP loci with the highest MAF, excluding those that had been selected to be QTL, were selected as markers and the test and candidate sibs were genotyped for these *m* markers.

### Estimation of SNP effects

Estimation of SNP effects followed the GS-BLUP model [1] for *n* phenotypes with the *m* marker loci:

(2) $$y=\sum_{j=1}^{m}x_{\mathrm{ij}}u_{j}+e$$

where **y** is a vector of phenotypes, *x*_*ij*_ is the standardized number of a randomly chosen reference allele (allele “1”) carried by animal *i* at the *j*^*th*^ marker locus*,* as described below, *u*_*j*_ is the effect of allele “1” at locus *j*, and **e** is a vector of random errors assumed to be distributed as *N*(0, *σ*_*e*_^2^**I**). The variance of each marker effect was assumed to be drawn from identical independent distributions with $\sigma_{i}^{2}=\frac{\sigma_{A}^{2}}{m}$. standardized number of “1” alleles was computed as:

(3) $$x_{\mathrm{ij}}=\frac{x_{ij1}^{*}-2p_{j}}{\sqrt{2p_{j}\left(1-p_{j}\right)}}$$

where *p*_*j*_ is the frequency of allele “1” at the *j*^*th*^ marker locus and *x*_*ij*1_^*^ is the number of “1” alleles carried by individual *i*.

The elements x_*ij*_ form the incidence matrix **X** and the vector of SNP effects **u** was estimated (**û**) from:

(4) $$\left[X^{T}X+\frac{\sigma_{e}^{2}}{\sigma_{i}^{2}}I\right]\left[\hat{u}\right]=\left[X^{T}y\right]$$

The EBV of candidate *i* was predicted by using their SNP genotypes and summing up their marker effects, as estimated using the test population, as:

(5) $$GEBV=X\hat{u}$$

Standardization of SNP covariates *x*_*ij*_ for the candidates was carried out using the same values of *p*_*j*_ as used for the test animals, i.e. the estimates of frequencies were obtained using both candidate and test sets.

### Statistics

Outputs of the base populations obtained from the simulation of the founder ancestors were stored. Each replicate had its own base population. Averages of 100 replicates of each breeding scheme with different scenarios were compared in terms of inbreeding, genetic gain, accuracy of selection and variance reduction generated in G2. Inbreeding coefficients were computed using G0 as the base population.

## Results

### Trend in genetic parameters

**Figure 1:**
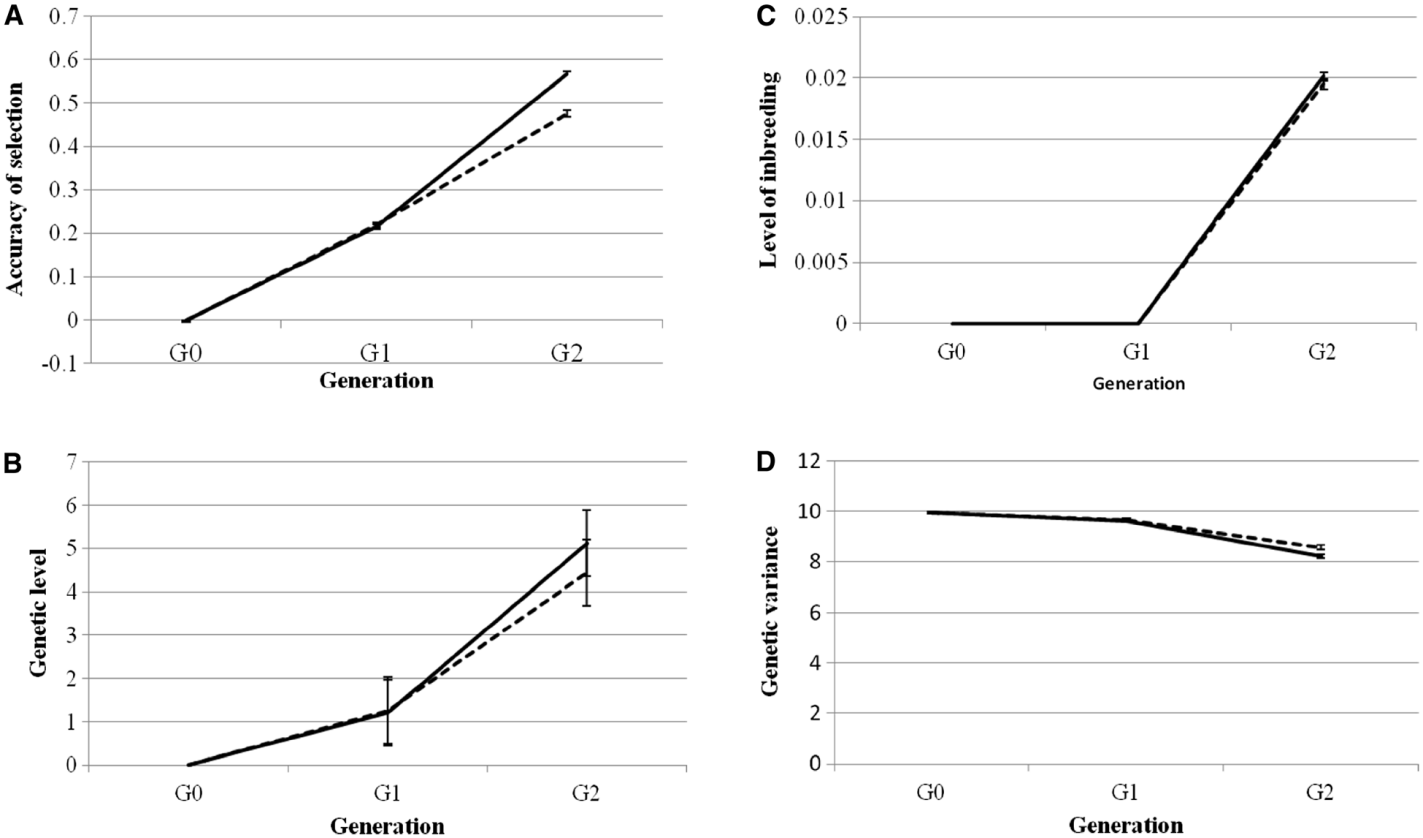
**Trends of genetic parameters in the candidate population.A**) Accuracy of selection. **B**) Genetic level. **C**) Level of inbreeding. **D**) Genetic variance. Trends of genetic parameters in the candidate population in the *MatPat* scheme (solid line): half of the test sibs were maternal double haploids and the other half were paternal double haploids in G2 and in the *RAND* scheme (dashed line): candidate and test sibs were mated at random in G1; in each scheme there were 1500 males and 1500 female candidate sibs, 50 sires and 50 dams were selected each generation and heritability was 0.05.

Figure 1 shows the trend in genetic parameters from G0 to G2 for the candidate population. Results are only shown for the *RAND* and *MatPat* schemes and h^2^ = 0.05 because of their extreme values, while with the other schemes intermediate values were obtained. More detailed results will be presented in the next section. In the base generation G0, the accuracy of selection (1A), genetic levels (1B) and levels of inbreeding (1C) were zero and genetic variance (1D) was 10. For all schemes, the onset of inbreeding was in G2. An increasing trend in genetic gain and accuracy of selection was observed from G1 to G2 and genetic variance decreased. Increases in genetic gain and selection accuracy and the reduction in genetic variance were greatest for the *MatPat* scheme.

### Genetic parameters in generation G2

#### Accuracy of selection

Accuracies of selection generated in the candidate population for all schemes are in Table 1. The highest accuracy of selection was obtained with the *MatPat* scheme, while with the *Pat* and *Mat* schemes it was lowest. As expected, *Pat* and *Mat* were always very similar in accuracy. A substantial increase in accuracy was observed for the *MatPat* scheme compared to the *RAND* scheme. For example, accuracy of selection increased by 19% for h^2^ = 0.05, by 12% for h^2^ = 0.1 and by 7% for h^2^ = 0.4. In contrast, use of a non-random mating scheme had only a small impact on accuracy and none of the differences were statistically significant (p > 0.05).

**Table 1:** Accuracy of estimated breeding values for the candidate population in G2

**Breeding scheme**	**h**^**2**^**= 0.05**	**h**^**2**^**= 0.1**	**h**^**2**^**= 0.4**
**Double haploid scheme**			
***MatPat***	0.568	0.642	0.767
***Pat***	0.463	0.524	0.639
***Mat***	0.469	0.527	0.638
**Diploid scheme**			
***MaxC***	0.481	0.569	0.720
***MinC***	0.474	0.567	0.724
***RAND***	0.476	0.571	0.720

Accuracy of estimated breeding values generated in G2 for the candidate population; standard errors of means of 100 replicates were less than 0.006; *MatPat*: the test sibs inherited maternal and paternal double haploid genomes in G2; *Pat*: all test sibs inherited paternal double haploid genomes in G2; *Mat*: all test sibs inherited maternal double haploid genomes in G2; *MaxC*: the test population was obtained by maximum coancestry mating; *MinC*: the test population was obtained by minimum coancestry; *RAND*: the candidate and test populations were obtained by random mating; in each scheme there were 1500 males and 1500 female candidate sibs, 50 sires and 50 dams were selected each generation and heritability was 0.05, 0.1 or 0.4.

#### Genetic gain

The genetic gains (**Δ***G*) generated in the candidate population are presented in Table 2 and on the whole they agree with the observed accuracies in Table 1. The highest **Δ***G* was achieved with the *MatPat* scheme, while the lowest **Δ***G* was obtained with the *Pat* and *Mat* schemes. Compared to the *RAND* scheme, all schemes had a statistically significant (p > 0.05) increase in **Δ***G* by 22% for h^2^ = 0.05, by 12% for h^2^ = 0.1 and by 6% for h^2^ = 0.4 (Table 2).

**Table 2:** Genetic gain generated in G2 in the candidate population

**Breeding scheme**	**h**^**2**^**= 0.05**	**h**^**2**^**= 0.1**	**h**^**2**^**= 0.4**
**Double haploid scheme**			
***MatPat***	1.23	1.40	1.58
***Pat***	1.00	1.12	1.32
***Mat***	1.01	1.12	1.33
**Diploid scheme**			
***MaxC***	1.03	1.22	1.49
***MinC***	1.06	1.22	1.49
***RAND***	1.01	1.25	1.49

Genetic gain in standard deviation units (**Δ**G) generated in G2 in the candidate population; standard errors of means of 100 replicates were less than 0.02; see Table 1 for explanation of schemes.

#### Level of inbreeding

Levels of inbreeding generated in the candidate population are in Table 3. As expected, level of inbreeding decreased as heritability increases. Compared to the diploids schemes, the differences in inbreeding attained appeared to be slightly higher in the double haploids schemes at lower heritability (h^2^=0.05). Thsese differences diminished as heritability increases. In addition, across all level of heritabilities, the level of inbreeding generated in the double haploid and diploid schemes were not significantly different (p > 0.05) from each other.

**Table 3:** Level of inbreeding generated in G2 in the candidate population

**Breeding scheme**	**h**^**2**^**= 0.05**	**h**^**2**^**= 0.1**	**h**^**2**^**= 0.4**
**Double haploid scheme**			
***MatPat***	0.201	0.187	0.147
***Pat***	0.208	0.181	0.136
***Mat***	0.214	0.189	0.141
**Diploid scheme**			
***MaxC***	0.192	0.190	0.150
***MinC***	0.194	0.189	0.152
***RAND***	0.195	0.178	0.153

Level of inbreeding in percentage generated in G2 in the candidate population; standard errors of means of 100 replicates were less than 0.006%; see Table 1 for explanation of schemes.

#### Variance reduction

The genetic variances generated in the candidate population are in Table 4. As a result of selection of parents in G1, the genetic variance was reduced by 15% to 30% in G2 compared to G0, depending on the scheme and heritability. Comparisons among the double haploid schemes show that the genetic variance retained was slightly higher in the *Mat* and *Pat* schemes and lower in the *MatPat* scheme. In contrast, the genetic variances retained within the diploid schemes were not significantly different (p > 0.05) from each other, and for higher heritabilities tended to be intermediate between the *MatPat* and the single sex double haploid schemes. Overall, the pattern of differences in genetic variances between heritabilities and schemes was qualitatively similar to the pattern observed for differences in accuracies (Table 1).

**Table 4:** Fraction of genetic variance retained in G2 in the candidate

**Breeding scheme**	**h**^**2**^**= 0.05**	**h**^**2**^**= 0.1**	**h**^**2**^**= 0.4**
**Double haploid scheme**			
***MatPat***	82	78	70
***Pat***	87	86	78
***Mat***	86	85	77
**Diploid scheme**			
***MaxC***	87	81	72
***MinC***	87	82	72
***RAND***	86	82	72

Fraction of genetic variance in percentage retained in G2 in the candidate population; standard errors of means of 100 replicates were less than 0.09%; see Table 1 for explanation of schemes.

## Discussion

It has been reported that genomic selection in aquaculture breeding schemes can increase selection accuracy for candidates and increase genetic gain compared to traditional aquaculture sib testing schemes [8,9,19]. Our study shows that, when using genomic selection, creating double haploids as part of the process of sib testing can increase the selection accuracy of candidates and the genetic gain even more. These additional increases in accuracy and genetic gain were most dramatic with a low heritability (~22%) but were still substantial when heritability was 0.4 (~7%). However, this result was only obtained when both sexes were used to create double haploids for testing. When only one sex is double haploid, selection accuracy was reduced because only chromosome sets from the dam (sire) entered the test population and this was not offset by increasing the number of observations per chromosome set.

In this study, attempting to increase homozygosity above that obtained from random mating through maximum coancestry mating when breeding the test population had no detectable impact on genetic gain or inbreeding. This is because assortative mating was only done for one generation, which is unlikely to produce extreme genotypes. If breeding of the test population with the *MaxC* scheme had been continued for 10 generations or more, similar results to those obtained with the double haploids scheme would have been observed because it takes approximately 10 generations of continuous full sib mating to produce fully inbred lines [14]. Clearly one generation of either *MaxC* or *MinC* is insufficient to deliver any benefit.

The increase in selection accuracy of the candidate population is due to the increase in accuracy of the estimates of SNP effects in the test population because the double haploids have more extreme genotypic values. It is important to note that the study design used here permits this inference because the non-random mating structure in the test population was not replicated in the candidate breeding population, which was always bred at random. Therefore, the increased predictive accuracy was due to the test design and not the results of differences in family structure amongst the candidates. For example, if *MinC* mating had been implemented in the candidate population, say for five generations, to improve the family structure, genetic effects would eventually have been better estimated and a substantial increase in selection accuracy might have occurred [20].

The benefit of the improved accuracy from generating extreme genotypes in the test population does come at a cost in robustness to the underlying genetic architecture. The design provides an estimate of *a*, i.e. half the difference between the genetic value of homozygotes. In this study, the allelic effects were simulated to be additive, so the estimates of an allelic substitution were not biased by the absence of heterozygotes. However, if the dominance deviation *(d)* is not equal to zero, the average effects obtained from homozygotes are biased by *d*(1-2*p*) where *p* is the minor allele frequency. This bias increases as *p* reduces. Most QTL are expected to have a low minor allele frequency, potentiating the bias. Presence of epistatic gene actions are expected to result in similar biases from estimations based on homozygotes only.

The advantage obtained with the *MatPat* scheme was achieved at a cost of reduced genetic variation in G2 compared to other schemes. However, the results show that this reduction in genetic variation was mainly generated by additional linkage disequilibrium [21] created by the higher accuracy obtained with the *MatPat* scheme. First, inbreeding accounted for only a loss of 2% of the genetic variance and any difference in inbreeding between *MatPat* with the other schemes was not substantial. Under the infinitesimal model, the loss of genetic variance due to linkage disequilibrium would be ½*ρ*^2^ where *ρ* is the accuracy of selection [21]. Thus with *ρ* = 0.6, predicted loss would be 18%. Using (1-½*ρ*^2^)(1-*F*) predicted losses in genetic variance observed in Table 4 from the accuracies of selection presented in Table 1 gives a very close approximation. Experience with the infinitesimal model has demonstrated that increasing selection intensity results in greater short and medium term genetic gain even though this also increases the linkage disequilibrium. However, this is not the case when selection is on a known or marked QTL along with estimates of unlinked polygenic effects, for which slowing fixation of the QTL has been shown to result in increased long-term gain [22]. The existence of a trade-off between accuracy and long-term gain that is independent of inbreeding (genomic or pedigree) has not been reported to date.

There are two methodological approaches to produce double haploids, either mitotic or meiotic. In this study, mitotic gynogenesis and mitotic androgenesis were used. There are good reasons to expect that the extra genetic gains would have been somewhat less with meiotic gynogenesis and meiotic androgenesis because these technologies result in fish that are less homozygous than their mitotic counterparts, i.e. only a subset of their genotypes are homozygous [15].

The increased accuracy of selection with use of a double haploid sib test population results from the explanatory variables in the regression equation (Equation 2) taking more extreme values due to inbreeding, which, based on regression theory [23] , is known to increase the accuracy of the estimation of SNP effects. As derived in the Appendix, the increase selection accuracy from the perspective of genomic relationships and selection index theory can be explained using the formula:

(6) $$E\left[r_{\mathrm{GS}}^{2}\right]=A_{21}^{T}P^{-1}A_{21}+trace\left(P^{-1}V\right)$$

Where **A**_21_ is the relationship between sib test individuals and one of the selection candidates, and **P** is a phenotypic covariance matrix for the sib test individuals, and **V** is the variance of relationships between the test sibs.

This shows the expected reliability (= squared accuracy) of genomic selection is approximately equal to the reliability of traditional EBV plus a term that increases with the variances of the deviations of genomic relationships from their expectations in the test population. The implications of this formula go further in defining conditions that maximize the expected reliability of genomic selection: (1) the training animals should be as little related as possible, which makes **P**^-1^ large; (2) the training animals should be as much related to the selection candidates as possible, which makes **A**_21_ large; and (3) deviations of the genomic relationships from the traditional relationships should be as large as possible, resulting in large **V**. Points (1) and (2) were also observed by [12]. Double haploids have the same expected relationship with the candidates as diploid training animals (½), but the variance of their genomic relationship with the candidates is increased due to inbreeding. Thus, the increase in accuracy of genomic selection when using double haploids can be explained in two ways: (1) the more extreme regression factors in (Equation 2) allow SNP effects to be more accurately estimated, or (2) their more variable relationships with the candidates can be used by selection indices and BLUP to increase the accuracy of the EBV of the candidates.

Here, GS-BLUP [1] was used for genetic evaluation. Similar outcomes in terms of increases in accuracy and genetic gain from double haploids and non-random mating are expected with other genomic evaluation methods that are currently used. For example, the *BayesB* method [1] concentrates on certain important regions of the genome. Double haploid individuals are also double haploids for these regions of the genome. Therefore, the variance of genomic relationships between individuals in the test and candidate individuals also increases at these regions of the genome and thus delivers a more accurate estimation of SNP effects than the test population produced by random mating.

It is expected that the use of double haploid sib test populations increases the accuracy of genomic selection for any candidate population because use of double haploids achieves a more accurate estimation of the SNP effects in the test population by increasing the variance of the genomic relationships between the test and candidate population. This is expected to hold for any candidate populations. However, the selection candidates should not be completely different from the test population

### Relevance of the study

This study shows the benefits of using double haploids as test sibs in aquaculture genomic selection breeding schemes. An increase of 7 to 19% in selection accuracy, leading to a 6 to 22% increase in genetic gain was obtained. This resulted from more accurate estimation of SNP effects and required a mixture of paternal and maternal double haploid test sibs in combination with genomic selection. Increases in accuracy and genetic gain from use of double haploids were greater with lower heritability levels. Therefore, the outputs of this study can be used to increase genetic gain for difficult traits such as disease resistance and meat quality, which cannot be recorded on selection candidates. In practical applications, eggs may be collected from the nucleus breeding parents and divided in two parts and 50% would be fertilized in a natural way, forming the candidate population, and the rest further divided into one half that is submitted to gynogenesis and the other half to androgenesis. This would result in a mixture of maternal and paternal double haploid genome fishes from each family in the test population.

However, there are practical problems to overcome for the implementation of double haploids in aquaculture breeding schemes, including biases from non-additive gene effects costs and other practical constraints. There may also be ethical and regulatory issues related to animal welfare associated with the use of chromosome manipulation techniques.

## Conclusions

This study has shown that the use of double haploids that produce inbred test sibs for estimation of SNP effects significantly increased selection accuracy and genetic gain. This required a mixture of test sibs with maternal and paternal double haploid genomes. The approach yielded increases in selection accuracy of up to 19% and in genetic gain of up to 22%. The double haploid technique produces inbred fish in one generation, which increased the accuracy of the estimation of SNP effects. Another strategy, in which the test population was designed based on non-random mating such as maximum coancestry mating, hardly improved selection accuracy. Finally, this study demonstrated the benefit of using distinct designs for the testing versus the candidate population.

## Appendix

### Genetic relationships and accuracy of selection

Based on selection index theory and assuming genetic variance is 1, the reliability (squared accuracy) of traditional selection is (assuming):

$$r^{2}=A_{21}^{T}P^{-1}A_{21}$$

where **A**_21_ is the relationship between sib test individuals and one of the selection candidates, and **P** is a phenotypic covariance matrix for the sib test individuals, i.e. **P** = **A**_22_ + **E**, where **A**_22_ is the pedigree-based relationship between the sib test individuals and **E** is a diagonal matrix of scaled environmental variances with (1-*h*^2^) / *h*^2^ on the diagonal. Let the genomic relationship matrices be denoted by **G**_22_ = **A**_22_ + **Δ** and **G**_21_ = **A**_21_ + **δ**, where **Δ** and **δ** represent changes from the expected relationships due to the genomic information. Then with genomic selection, the squared accuracy of selection is:

$$\begin{array}{ll} r_{\mathrm{GS}}^{2}\; & ={\left(A_{21}+\delta \right)}^{T}{\left(P+\Delta \right)}^{-1}\left(A_{21}+\delta \right)\\ & =A_{21}^{T}{\left(P+\Delta \right)}^{-1}A_{21}+\delta^{T}{\left(P+\Delta \right)}^{-1}\delta \end{array}$$

assuming that **A**_21_ and **δ** are independent.

Since (**P** + **Δ**) = **P**(**I** + **P**^− **1**^**Δ**), (**P** + **Δ**)^− **1**^ ≈ **P**^− **1**^ − **P**^− **1**^**ΔP**^− **1**^ then: ,

$$\begin{array}{ll} r_{\mathrm{GS}}^{2} & \;={\left(A_{21}+\delta \right)}^{T}{\left(P+\Delta \right)}^{-1}\left(A_{21}+\delta \right)\\ & \;=A_{21}^{T}P^{-1}A_{21}+\delta^{T}P^{-1}\delta -\delta^{T}P^{-1}\Delta P^{-1}\delta \end{array}$$

assuming the **A**_**21**_^**T**^**P**^− **1**^**ΔP**^− **1**^**A**_**21**_ term is on average 0, since **Δ** is on average 0. Taking expectations:

$$\begin{array}{ll} E\left[r_{\mathrm{GS}}^{2}\right] & =A_{21}^{T}P^{-1}A_{21}+E\left[\delta^{T}P^{-1}\delta \right]\\ & -E\left[\delta^{T}P^{-1}\Delta P^{-1}\delta \right] \end{array}$$

where the term *E*[**δ**^**T**^**P**^− **1**^**δ**] = *trace*(**P**^− **1**^**V**), where **V** = var(**δ**). The third term

$$\begin{array}{ll} E\left[\delta^{T}P^{-1}\Delta P^{-1}\delta \right] & =E_{\Delta }\left[E\left[\delta^{T}P^{-1}\Delta P^{-1}\delta |\Delta \right]\right]\\ & =E_{\Delta }\left[trace\left(\Delta P^{-1}VP^{-1}\right)\right]\\ & =0,since\;E\left[\Delta \right]=0\text{.} \end{array}$$

Thus,

$$E\left[r_{\mathrm{GS}}^{2}\right]=A_{21}^{T}P^{-1}A_{21}+trace\left(P^{-1}V\right)$$

## Competing interests

The authors declare that they have no competing interests.

## Authors’ contributions

All authors were involved in the design of the study. KGN wrote the draft manuscript and ran the computer programs. THEM and AKS wrote simulation computer programs. AKS, THEM and JAW edited the drafted manuscript. All authors have read and approved the final manuscript.
